# On the Prediction of pDNA Productivity Across Diverse Bioprocesses Using Ensemble Hybrid Models

**DOI:** 10.1002/bit.70207

**Published:** 2026-05-13

**Authors:** Nikolaos Stratis, Massimo Morbidelli, Alexandros Kiparissides

**Affiliations:** ^1^ Department of Chemical Engineering Aristotle University of Thessaloniki Thessaloniki Greece

**Keywords:** artificial neural networks, hybrid models, pDNA, process development, transfer learning

## Abstract

The global demand for plasmid DNA (pDNA) is rapidly increasing due to its key role in the manufacture of mRNA vaccines and advanced therapy medicinal products (ATMPs). However, the reliance on suboptimal batch and/or fed‐batch processes, as well as the diversity and increasing complexity of plasmid constructs pose significant challenges during process development. In fact, the diversity of manufactured plasmids and employed host cell lines diminishes the benefits of using traditional model‐based approaches to expedite process development despite the availability of sufficient data. To address these limitations, an ensemble hybrid modeling approach that integrates mechanistic dynamic mass balances with data‐driven deep learning derived kinetic rates was developed. Briefly, each rate term was replaced with a fully connected artificial neural network (ANN) whose input variables included a vector of all state variables at timepoint (*t*), *H* historical data vectors of all first‐order derivatives at timepoints [*t*–*H* and *t*–1] and one‐hot‐encoded categorical variables (host, plasmid, media, and mode of operation). Hybrid models were trained and validated using an 80/20 ratio on a literature derived dataset comprising data from 18 unique *E. coli* fermentations across different media formulations, different plasmids, and different culture modes. Subsequently, a progressive transfer learning framework was employed to assess adaptability of a pretrained model to unseen *E. coli* strains and culture modes (from batch to fed‐batch). This approach exploited historical data to substantially reduce model recalibration costs, emphasizing the potential of hybrid and transfer learning methods to accelerate pDNA bioprocess development.

## 1 Introduction

The rapid expansion of cell and gene therapies, mRNA and DNA vaccines has transformed pDNA into a valuable commodity for the biopharmaceutical industry. The global annual demand for pharmaceutical grade pDNA is currently projected to surpass 100 kg by 2030 (Triantafyllou et al. 2024). pDNA is typically produced in batch or fed‐batch *Escherichia coli* fermentations at pilot (30–50 L) or industrial scale (1000–4000 L) with titers usually below 1 g/L. While K12 derived *E. coli* strains have dominated the biopharmaceutical industry, several variants exist (DH5α, DH10B, JM109, and W3310) with additional multiple‐deletion series strains under development (Gonçalves et al. 2014). The existence of several potential host cell lines, combined with the increasing diversity of manufactured plasmids and the often unpredictable nature of host‐plasmid interactions poses substantial time and cost intensive challenges during process development.

This diversity also hinders the use of mathematical modeling‐based approaches to guide and expedite process development as has been successfully done for other modalities (Sulieman et al. 2018; Carranza‐Saavedra et al. 2021). Therefore, despite the existence of multiple public and/or proprietary batch record datasets, only a few mechanistic models of pDNA bioproduction kinetics can be found in the literature (Lopes et al. 2014). Batch record data remain one of the most underutilized resources in the biopharmaceutical industry, primarily due to the lack of appropriate numerical approaches able to extract value from diverse and often inconsistent data (Helleckes et al. 2023). Recent advances in deep learning, particularly in the field of Artificial Neural Networks (ANNs), have enabled the integration of increasingly complex nonlinear relationships into data‐driven modeling frameworks. Therefore, what is presently missing are robust modeling approaches that can utilize the wealth of available batch record data to guide future process design efforts by efficiently minimizing the amount of new data and/or experiments required.

Appropriately constructed mechanistic (or “first‐principles”) models based on fundamental physical and biological principles can accurately represent the system state at any given time (Lopes et al. 2014). However, development and parametrization of such models is labor intensive and parameter values do not generally transfer between diverse processes (e.g., when considering different cell lines or culture modes) (Rogers et al. 2022). Modern, data‐driven approaches on the other hand can handle data heterogeneity, whether in categorical or continuous variables, but suffer from limited extrapolation capacity, lack interpretability, and may violate fundamental biophysical constraints (Bayer et al. 2023; Mahanty 2023; Helleckes et al. 2023; Pinto et al. 2019). In response, hybrid modeling approaches have emerged in an attempt to mitigate the respective limitations of both purely mechanistic and purely data‐driven approaches (Bayer et al. 2023; Mahanty 2023; Helleckes et al. 2023; Pinto et al. 2019; von Stosch et al. 2016). This is achieved by employing rigid mechanistic structures, such as mass balances, to ensure compliance with physical laws and limitations and augmenting them with machine learning predictors, usually of rates and/or states.

Herein, the development of a hybrid modeling framework to predict pDNA production kinetics across a variety of strains, plasmids, and culture modes is presented. Initially, a comprehensive and diverse dataset of pDNA producing *E. coli* cultures was curated from the scientific literature. The dataset comprises 18 unique experimental conditions, spanning different media formulations, different plasmid constructs, and even different culture modes. In order to cope with the diverse nature of the dataset, ANN‐based kinetic rate estimators were integrated with a mechanistic model chassis comprised of dynamic mass balance equations. Briefly, each rate term was replaced with an Artificial Neural Network (ANN) whose input variables included a vector of all state variables at timepoint (*t*), *H* historical data vectors of all first‐order derivatives at timepoints [*t*–*H* and *t*–1] and one‐hot‐encoded categorical variables (host, plasmid, media, and culture mode). To avoid overfitting an ensemble method was employed, while the dataset was split into training and validation datasets with an 80/20 ratio. The developed hybrid model was able to predict the outcomes of vastly diverse culture conditions with acceptable accuracy. Finally, several transfer learning strategies were evaluated to assess the model's adaptability to new host cell lines and fermentation modes.

This study highlights the potential of hybrid deep‐learning modeling frameworks to accelerate process development and reduce experimental workload in pDNA manufacturing. Such frameworks can streamline experimental design and facilitate rapid optimization, ultimately enhancing efficiency in plasmid bioproduction.

## 2 Materials and Methods

All simulations were performed using Python (v3.8.18) (Paszke et al. 2019; Python Software Foundation 2023) and the algorithms included in the PyTorch library (Paszke et al. 2019).

### 2.1 Data Curation

Experimental measurements from 18 pDNA producing *E. coli* fermentations were retrieved from the scientific literature. The compiled dataset, summarized in Table 1, contains data from three distinct *E. coli* strains (DH5a, VH33, and W3310), two distinct plasmids (pVAX‐Lacz and pcDNA3.1), two types of media (complex and defined), two carbon sources (glucose and glycerol), two pH regimes (7.0 and 7.2), and two modes of operation (batch and fed‐batch). The complete dataset is available in Supporting Material S5.

**Table 1:** Overview of datasets used for model development. Primary carbon sources are denoted as Glc for glucose and/or Gly for glycerol. Presence (+) or absence (–) of antibiotic in the media is denoted by the respective symbol.

Exp.	Strain	Plasmid	Media	pH	Mode	Source
1	DH5a	pVAX‐LacZ	Complex/Glc (−)	7.0	Batch	Lopes et al. (2014)
2	DH5a	pVAX‐LacZ	Complex/Gly (−)	7.0	Batch	Lopes et al. (2014)
3	DH5a	pVAX‐LacZ	Complex/Glc, Gly (−)	7.0	Batch	Lopes et al. (2014)
4	DH5a	pVAX‐LacZ	Complex/Glc, Gly (−)	7.0	Batch	Lopes et al. (2014)
5	DH5a	pVAX‐LacZ	Complex/Glc, Gly (−)	7.0	Batch	Lopes et al. (2014)
6	DH5a	pcDNA3.1	Defined/Glc (+)	7.2	Batch	Borja et al. (2012)
7	DH5a	pcDNA3.1	Defined/Glc (+)	7.2	Batch	Borja et al. (2012)
8	VH33	pcDNA3.1	Defined/Glc (+)	7.2	Batch	Borja et al. (2012)
9	VH33	pcDNA3.1	Defined/Glc (+)	7.2	Batch	Borja et al. (2012)
10	VH33	pcDNA3.1	Defined/Glc (+)	7.0	Batch	Soto et al. (2011)
11	W3310	pcDNA3.1	Defined/Glc (+)	7.0	Batch	Soto et al. (2011)
12	W3310	pcDNA3.1	Defined/Glc (+)	7.0	Batch	Soto et al. (2011)
13	VH33	pcDNA3.1	Defined/Glc (+)	7.0	Batch	Soto et al. (2011)
14	DH5a	pVAX‐LacZ	Complex/Gly (−)	7.0	Batch	Sales et al. (2015)
15	VH33	pcDNA3.1	Defined/Glc (+)	7.0	Fed‐batch	Soto et al. (2011)
16	DH5a	pcDNA3.1	Complex/Glc (−)	7.0	Fed‐batch	Ow et al. (2009)
17	DH5a	pVAX‐LacZ	Complex/Glc (−)	7.0	Fed‐batch	Sales et al. (2015)
18	DH5a	pVAX‐LacZ	Complex/Glc, Gly (−)	7.0	Fed‐batch	Sales et al. (2015)

**Figure 1:**
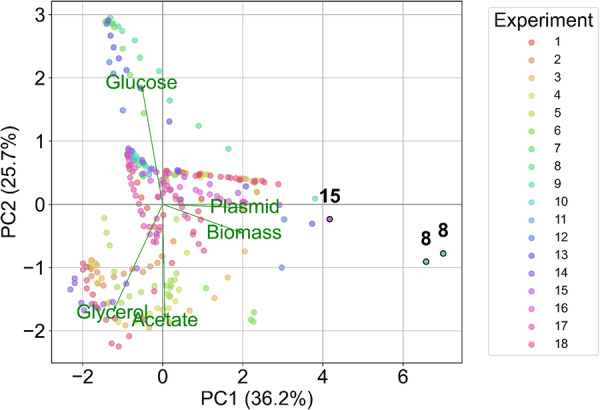
Principal component analysis (PCA) biplot of experimental data points. Vectors represent the loadings of key state variables (glucose, glycerol, acetate, biomass, and plasmid concentrations). Data points are colored according to their corresponding experiments.

Principal component analysis (PCA) was used to assess variability in the dataset and identify potential outliers based on z‐scores (Sawant et al. 2012). Only three data points exhibited z‐scores greater than 3 (labeled points in Figure 1), a common threshold used for outlier detection (Venkataanusha et al. 2019; Paszke et al. 2019). However, these points correspond to physiologically relevant conditions (high initial glucose concertation and fed‐batch operation) and were therefore retained in the analysis. The first two principal components captured more than 60% of the total variance (PC1 = 36.2% and PC2 = 25.7%) and the calculated loadings captured correlations consistent with biological and process knowledge increasing confidence in the quality of the data. The loadings for glucose and acetate concentrations were oriented in opposite directions, highlighting that acetate accumulation is concurrent with glucose depletion, an indication of overflow metabolism commonly observed under high substrate concentrations (Pinto et al. 2019; Zeng and Yang 2019). The loading for pDNA concentration was closely aligned with the loading for biomass concertation indicating a strong correlation but in contrast perpendicular to the loading for acetate concentration, implying no correlation between the two variables.

Finally, cubic spline interpolations were used to augment the data for each measured state variable across each experiment used for model training to 100 data points per trajectory. This was done to avoid bias by ensuring a balanced representation across all experiments and measured state variables. While cubic spline interpolation is an established data augmentation technique which minimizes artificial discontinuities that can negatively affect training, the use of artificial data, even if interpolated, can introduce bias and obfuscate true process behavior. Both the choice of augmentation method and the extent of its use (ratio of real to interpolated data points) are important aspects of model development which should be further explored in order to elucidate their effect on the quality of model predictions (Mat Leh et al. 2024; Ramos et al. 2024; Oh et al. 2020).

### 2.2 The Knowledge‐Driven Model Chassis

Dynamic mass balance equations (Table 2) were formulated for key process variables based on the availability of experimental data. Rate terms were introduced following the rationale presented in Lopes et al. (Lopes et al. 2014). Consequently, the dynamic mass balance for acetate (Equation 1, Table 2) contains both a specific consumption (*r_A_*) and specific production (*r_PA_*) rate term. The use of two separate rates while consistent with established descriptions of overflow metabolism (Corrao et al. 2025; Millard et al. 2021), introduces significant identifiability concerns since the available experimental data provide direct measurements of acetate concentration. Consequently, only a net observed rate of change can be reliably derived from the experimental data. Nevertheless, in the present work, the original model structure of Lopes et al. (Lopes et al. 2014) was preserved to retain the mechanistic interpretability in the hybrid model formulation. Therefore, herein the two rate terms function as internal model components that encode prior process knowledge, whereas the net acetate dynamics remain the directly primary observed state. For fed‐batch experiments, inlet flowrates (*F*_in_) were explicitly calculated from the reported experimental protocols (Supporting Material S1) (Soto et al. 2011; Ow et al. 2009).

**Table 2:** Dynamic mass balance equations.

Variable	Symbol [units]	Equation	
Biomass concentration	X $\left[\frac{g_{\mathrm{DCW}}}{L}\right]$	$\frac{\mathrm{dX}}{\mathrm{dt}}=\;r_{X}\cdot X-\frac{X}{V}\cdot \frac{\mathrm{dV}}{\mathrm{dt}}$	(1)
Glucose concentration	S $\left[\frac{g}{L}\right]$	$\frac{\mathrm{dS}}{\mathrm{dt}}=\;{-r}_{s}\cdot X+\frac{S_{\mathrm{in}}}{V}\cdot F_{\mathrm{in}}-\frac{S}{V}\cdot \frac{\mathrm{dV}}{\mathrm{dt}}$	(2)
Glycerol concentration	G $\left[\frac{g}{L}\right]$	$\frac{\mathrm{dG}}{\mathrm{dt}}=\;{-r}_{G}\cdot X+\frac{G_{\mathrm{in}}}{V}\cdot F_{\mathrm{in}}-\frac{G}{V}\cdot \frac{\mathrm{dV}}{\mathrm{dt}}$	(3)
Acetate concentration	A $\left[\frac{g}{L}\right]$	$\frac{\mathrm{dA}}{\mathrm{dt}}=(-r_{A}+r_{\mathrm{PA}})\cdot X-\frac{A}{V}\cdot \frac{\mathrm{dV}}{\mathrm{dt}}$	(4)
pDNA concentration	P $\left[\frac{\mathrm{mg}}{L}\right]$	$\frac{\mathrm{dP}}{\mathrm{dt}}=\;r_{P}\cdot X-\frac{P}{V}\cdot \frac{\mathrm{dV}}{\mathrm{dt}}$	(5)
Volume	V [L]	$\frac{\mathrm{dV}}{\mathrm{dt}}=\;F_{\mathrm{in}}-F_{\mathrm{out}}$	(6)

### 2.3 The Data‐Driven Kinetic Rate Predictors

Each rate term in Equations 1–6 (Table 2) was replaced with an ANN which receives three distinct input “modules,” one representing the present state, one representing the (recent) past, and one containing categorical information. Information about the present values for all state variables at time point (*t*) can be compiled in a 5 × 1 vector (Equation 8). Historical information about each state's (recent) past is captured through the (*H*) most recent values (*t*–*H* and *t*–1) of its first‐order derivative (*dx*/*dt*), where (*H*) is a tunable hyperparameter (Equation 9). The derivatives are obtained from model‐predicted state trajectories following smoothing spline reconstruction to avoid amplification of potential noise. This results in a 5 × H matrix, when considering all five state variables, which is subsequently flattened to 5H × 1 for input compatibility. Finally, information about categorical variables is provided through a one‐hot encoding (OHE) vector. Five categorical variables have been considered in the present work, including host strain, plasmid type, media type, pH set‐point, and primary carbon source (Table 3). Each category is assigned a fixed position in the OHE vector as detailed in Table 3. The corresponding element in the OHE vector is assigned a value of 1 if the category is active/true and 0 otherwise. The high‐level structure of the ANN rate predictors can be therefore summarized by Equations 7–9:

(7) $${\hat{r}}_{t}=f_{\theta }\left({\vec{x}}_{t},{\vec{\mathrm{dx}}}_{H},{\vec{v}}_{\mathrm{OHE}}\right)$$

where:

(8) $${{\vec{x}}_{t}=[X_{t},S_{t},G_{t},A_{t},P_{t}]}^{T}$$

(9) $${\vec{\mathrm{dx}}}_{H}=\mathrm{flatten}\left(\begin{array}{rcl} {\left.\frac{\mathrm{dX}}{\mathrm{dt}}\right|}_{t-1} & \cdots & {\left.\frac{\mathrm{dP}}{\mathrm{dt}}\right|}_{t-1}\\ \vdots & \ddots & \vdots \\ {\left.\frac{\mathrm{dX}}{\mathrm{dt}}\right|}_{t-H} & \cdots & {\left.\frac{\mathrm{dP}}{\mathrm{dt}}\right|}_{t-H} \end{array}\right)$$

**Table 3:** One‐hot encoding of categorical variables.

Categorical variables	Categories	OHE vector position
Strain	DH5α	1
	VH33	2
	W3310	3
Plasmid	pVAX‐LacZ	4
	pcDNA 3.1	5
Media	Complex	6
	Defined	7
pH	7.0	8
	7.2	9
Carbon source	Glucose	10
	Glycerol	11
	Glucose + Glycerol	12

Detailed information about the ANN architecture can be found in Supporting Material S2. Briefly, the network initially employs a normalized linear layer to map inputs to the hidden dimension. The hidden dimension comprises rectified linear unit (ReLU) activation functions embedded within linear layers, forming nested linear‐ReLU blocks. The outer layer uses a softplus function embedded within a linear layer to ensure strictly positive predictions and smooth behavior for small input values, promoting stable learning. Bayesian optimization was employed to tune ANN hyperparameters (Supporting Material S2). The search targeted the number (*H*) of historical derivative values provided as input (${\vec{\mathrm{dx}}}_{H}$), as well as the number of hidden layers and neurons in the network, evaluated across 50 sequential iterations to balance convergence and computational cost.

Despite significant research, a definitive consensus on the optimal neural network (NN) architecture for bioprocess state prediction has not yet been established. In the present work, design choices were guided by prioritizing prediction robustness over absolute goodness‐of‐fit. The employed dataset contains sparse and heterogeneous data across diverse operating conditions, factors that typically lead to high parameter estimation variability (or reduced confidence) during training. To mitigate this, an ensemble modeling approach was employed where multiple independently trained networks were used to derive an aggregate prediction, leading to improved robustness and prediction stability (Liu and Gunawan 2017). However, while advanced architectures such as Recurrent Neural Networks (RNNs) and Long Short‐Term Memory networks (LSTMs) are highly effective for capturing temporal dependencies, their training overhead becomes a limiting factor within an ensemble framework, where computational resources must be balanced against ensemble depth (Ramos et al. 2024). To maintain a robust number of ensemble members, a Feed‐Forward (FNN) architecture was selected. To compensate for the FNN's lack of intrinsic memory, historical process data in the form of the (*H*) most recent first‐order derivatives (*dx*/*dt*) were provided as network inputs. Derivative information was prioritized over raw state values to align the NN's learning objective with kinetic rates rather than states, ensuring that dynamic metabolic transitions are explicitly captured via gradient profiles. While the authors acknowledge that further methodological refinements remain possible particularly as the field evolves, the present work demonstrates the robust predictive potential of ensemble‐based hybrid models in complex bioprocessing landscapes.

Hybrid model performance was evaluated using a custom loss function designed to generate predictions that fit experimental data, but also ensure smooth dynamics to prevent overfitting. The loss function (Equation 10) comprises a relative root mean squared error (rRMSE) term combined with a term that penalizes large values in the second‐order derivative thereby enforcing smoothness and penalizing overfitting:

(10) $$L=\frac{\sqrt{\sum_{i=1}^{N}{\left(x_{i}-\hat{x_{i}}\right)}^{2}}}{\sigma_{\hat{x}}}+\lambda_{\mathrm{smooth}}\int {||\frac{d^{2}x}{dt^{2}}||}^{2}dt$$

where $x_{i}$ are model predicted values, ${\hat{x}}_{i}$ are experimental values, $\sigma_{x}$ is the experimental standard deviation of state variable i, N is the number of data points for state variable *i*, and $\lambda_{\mathrm{smooth}}$ is a scaling factor set equal to 1e^−3^ to balance the contribution of the goodness‐of‐fit and smoothness terms in the loss function. The second‐order derivative penalty is included as a regularization component to discourage highly oscillatory solutions and improve generalization. Second‐order derivatives are numerically approximated using central finite differences with step size equal to the integration time step *h* used by the Euler discretization scheme, ensuring consistency with the state propagation grid (Eilers and Marx 1996):

(11) $$\frac{d^{2}x_{i}}{dt_{i}^{2}}\approx \frac{x_{i+1}-2x_{i}+x_{i-1}}{h^{2}}$$

### 2.4 Ensemble Modeling

To enhance model robustness and provide quantitative uncertainty estimates, an ensemble modeling approach was employed. Ensemble modeling approaches are able to capture a broader spectrum of the underlying system dynamics while offering valuable insights into prediction reliability across the temporal domain (Pinto et al. 2019). The available experimental datasets were partitioned into training, validation, and test sets with a ratio of 60:20:20, respectively. This partitioning ensures comprehensive model training while maintaining sufficient data for unbiased performance evaluation. The test sets were manually selected to ensure that they did not introduce categories or concentration ranges absent from the training data. A combinatorial cross‐validation scheme was implemented for model tuning data sets (training and validation) to maximize data utilization and model diversity. The number of unique validation splits ($N_{\text{splits}}$) was determined by calculating the total number of combinations of *k* experiments chosen from the *n* available tuning experiments while respecting the 60:20:20 ratio:

(12) $$N_{\text{splits}}=\frac{n!}{k!(n-k)!}$$

The combinatorial formula generates all possible unique combinations of validation sets, ensuring that each split configuration is distinct and comprehensively explores the dataset's variability. By training across all cross‐validation folds, M unique, individually parametrized models are obtained. Fixed initial ANN weights were used across all models in the ensemble to avoid potential unintended variability on the models' convergence. The ensemble prediction for any state variable x at time t is computed as the mean of all individual model predictions, while prediction uncertainty can be quantified via the standard deviation:

(13) $$x_{t}=\frac{1}{M}\sum_{m=1}^{M}x_{t,m}$$

(14) $$\sigma_{t}=\frac{1}{M}\sqrt{\sum_{m=1}^{M}{\left(x_{t,m}-{\overset{ˉ}{x}}_{t}\right)}^{2}}$$

### 2.5 Transfer Learning

To evaluate the model's ability to generalize across different experimental domains, a transfer learning approach was applied. The objective was to assess the model's capacity to adapt to new conditions and previously unseen fermentation regimes whilst minimizing the amount of additional experimental data required for model recalibration. This approach was used to examine two industrially relevant scenarios: (i) transferability of historical process data to a new host cell line and (ii) transferability of historical batch process data to fed‐batch conditions.

For transferability across different host cell lines, experiments, including the VH33 cell line (Exp. 8, 9, 10, and 13; Table 1) were excluded from the training and validation sets. Similarly for transferability across process conditions, fed‐batch experiments (Exp. 15–18) were excluded from the training and validation sets. Ensemble hybrid models were tested against unseen experimental data after initial training and validation. Subsequently, the models were retrained by progressively adding experimental datasets either involving the VH33 cell line or from fed‐batch cultures, respectively. Two well‐established transfer learning strategies were evaluated to cover adaptation scenarios: (i) re‐estimation of only the outer layer parameters, which preserves previously learned internal representations while adapting the output mapping and (ii) fine‐tuning of all network parameters at a substantially reduced learning rate (one order of magnitude smaller than the original learning rate) to limit a potential complete parameter reconfiguration and reduce overfitting risk. These two transfer learning strategies were selected as representative baseline approaches in the absence of a consensus method for these types of problems. In both cases retraining was performed for 20 times less epochs resulting in substantially reduced computational costs. This procedure aimed to evaluate how efficiently the model could adapt to unseen process conditions while retaining the general features learned from the original dataset. Independent hybrid models were trained in parallel exclusively on VH33 or fed‐batch experiments as control. By comparing the performance of the re‐trained models against the respective *de novo* models, the extent at which prior knowledge can be transferred was quantified. Therefore, the potential benefits of transfer learning approaches could be assessed.

## 3 Results and Discussion

Historical data, whether from batch records, process development, or literature, are an invaluable yet underutilized resource. Up until recently, available mathematical modeling approaches were not able to satisfactorily capture such diverse datasets. Toward that aim, a series of ensemble hybrid models has been developed and assessed based on their ability to provide robust predictions in increasingly complex settings. Specifically, ensemble hybrid models were initially developed to accurately capture a diverse set of batch culture fermentations, including different host cell lines, different propagated plasmids, and different media formulations. Subsequently, transfer learning methodologies were applied to assess whether pre‐trained hybrid models can be adapted to unseen process conditions with a minimal amount of additional experiments.

### 3.1 Prediction of Diverse Batch Culture Data

**Figure 2:**
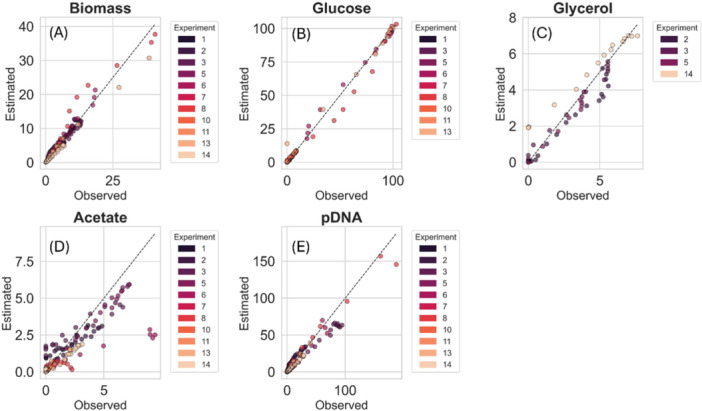
Hybrid model quality of fit on training and validation data. Model estimated (Y‐axis) vs. experimental (X‐axis) values for: (A) biomass, (B) glucose, (C) glycerol, (D) acetate, and (E) pDNA concentrations across all time points.

**Figure 3:**
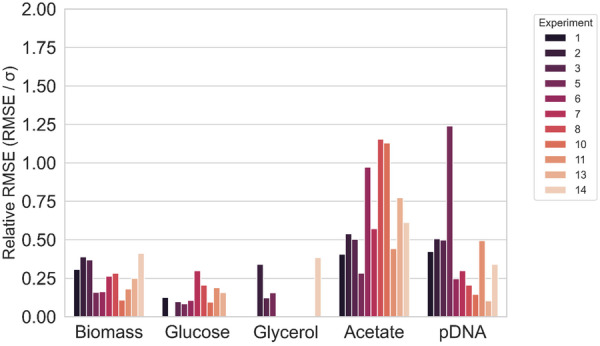
Hybrid model relative RMSE (rRMSE) across all training datasets.

Ensemble hybrid models were trained on data from 11 experimental datasets (Exp. 1, 2, 3, 5, 6, 7, 8, 10, 11, 13, and 14; Table 1) and tested against data from three experimental datasets (Exp. 4, 9, and 12; Table 1). Quality of fit to the training and validation sets was assessed through regression analysis (Figure 2) and estimation of relative RMSE (Figure 3). Overall, the ensemble hybrid models were able to fit the training data with low error (Figures 2 and 3) confirming the model's capacity to capture complex, non‐linear dynamics across diverse conditions. Interestingly, acetate concentration proved to be the most difficult state variable to capture (Figure 3). The temporal evolution of acetate concentration depends on host strain, carbon availability, plasmid characteristics, medium composition, and culture conditions (Lopes et al. 2014; Han and Eiteman 2019). This leads to highly varied trajectories as can be observed even within the limited datasets considered in the present study (Table 1, Supporting Material 3). Therefore, the hybrid model had difficulties capturing acetate concentration both during training and testing. pDNA concentration was generally well captured with the exception of Exp. 5 (Figure 3). Although experiments 1–5 (Table 1) involved the same host strain and plasmid, Exp. 5 exhibited higher acetate accumulation (7.2 $\frac{g}{L}$) which indicates highly active overflow metabolism. This inefficient use of metabolic resources could lead to limited resource availability for pDNA synthesis (Lopes et al. 2014). On the other hand carbon source consumption and biomass concentration were generally in very good agreement with experimental data, despite the considerable diversity of media formulations and host cell lines included in the data set.

**Figure 4:**
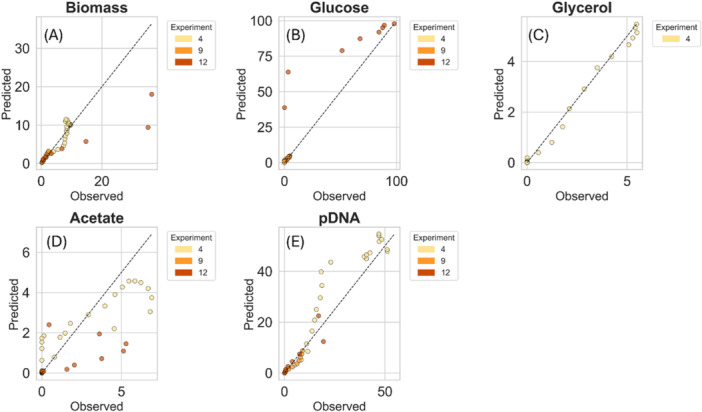
Hybrid model prediction quality on unseen data. Model predicted (Y‐axis) vs. experimental (X‐axis) values for: (A) biomass, (B) glucose, (C) glycerol, (D) acetate, and (E) pDNA concentrations across all time points.

**Figure 5:**
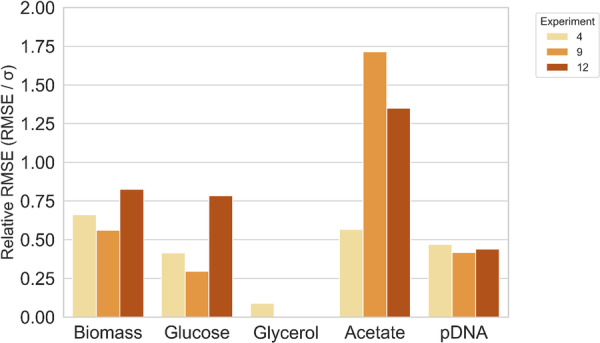
Hybrid model relative RMSE (rRMSE) of prediction across all unseen (test) datasets.

**Figure 6:**
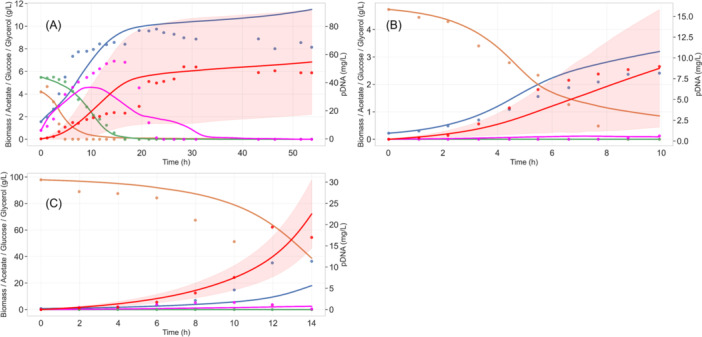
Simulated (–) and experimental (•) values for all state variables across all unseen datasets: (A) Exp. 4, (B) Exp. 9, and (C) Exp. 12. Data for biomass concertation (blue), glucose concentration (light orange), glycerol concentration (green), acetate concentration (magenta), and pDNA concentration (red, secondary Y‐axis). The shaded region represents ±1 standard deviation from the ensemble mean. For visual clarity, prediction uncertainty is shown only for this variable.

The same overall trends were carried over to the test set data, with acetate concentration remaining the most difficult state variable to capture (Figures 4 and 5). As expected, on average rRMSE values increased across all variables apart from, crucially, pDNA concentration (Figure 5). A notable deviation was observed in the late‐phase prediction of biomass concentration for Exp. 4 (Figure 6A). This can be attributed to the fact that all training and validation experiments had substantially shorter culture durations, preventing the model from learning long‐term stationary phase behavior. Biomass and acetate concentrations proved particularly difficult to predict for Exp. 12 (Figure 6C). This experiment was conducted at an initial glucose concentration of 100 g/L, which is substantially higher when compared to most of the other experiments in the dataset. In fact, the training set contained only three experiments under similar conditions (Exp. 8, 11, and 13; Table 1) which proved not to be enough for the model to be able to generalize in this specific regime. Nevertheless, pDNA concentration, which is the primary variable of interest, was accurately predicted across all test conditions.

### 3.2 Transfer Learning: Predicting Batch Process Performance for New Strains

**Figure 7:**
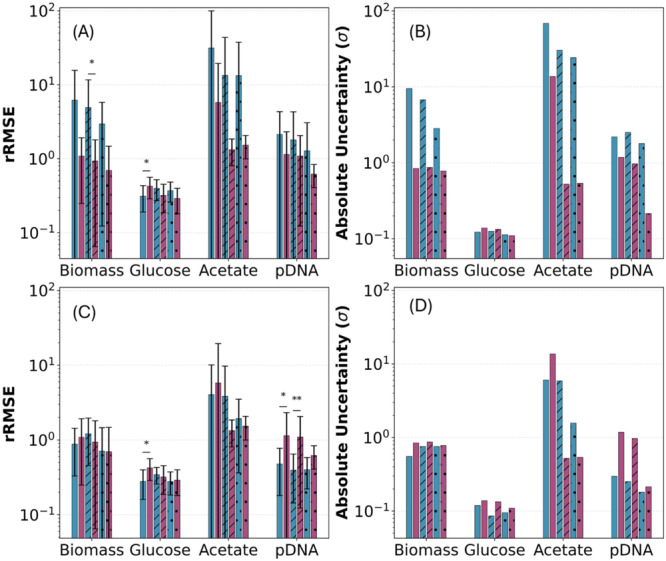
Prediction quality for a “new” unseen strain. (A and C) Mean relative RMSE (rRMSE) and (B and D) absolute uncertainty of prediction using a: (i) transfer learning approach with an ensemble hybrid model pretrained on different strains (blue bars) and (ii) a *de novo* ensemble hybrid model trained only on the “new” strain (purple bars). Bar patterns represent the number of experiments used for model (re‐) training which can be one (clear bars), two (dashed bars), or three (dotted bars) experimental datasets. Statistical comparisons conducted via Mann–Whitney U tests with asterisks denoting significant differences (*p < 0.05 and **p < 0.01). Transfer learning applied via (A and B) retraining the last layer in the ANN and (C and D) retraining all layers of the ANN at a reduced learning rate.

Migration to a new strain or even to a modified version of an existing cell line is a time and resource intensive procedure that often leads to substantial process (re‐) development efforts. Traditionally, model‐based analyses have followed the paradigm of re‐calibrating the model (at best) or completely redeveloping a model for the new strain or cell line (Helleckes et al. 2024; Hutter et al. 2021), which is data and time intensive. In the following section, the ability of transfer learning approaches to reduce both the wet‐lab and computational requirements of such modifications to the process is evaluated. An ensemble hybrid model was developed exclusively with batch culture experiments involving the DH5a strain (Exp. 1–7 and 14; Table 1). Subsequently, the model's ability to predict an unseen batch experiment involving the VH33 strain (Exp. 8–10 and 13; Table 1) when (i) the outer‐layer of the ANN was fine‐tuned (Rogers et al. 2022) (Figure 7A,B) or (ii) the entire ensemble was finetuned at a lower learning rate (Figure 7C,D) with 1, 2, or 3 additional “new” VH33 experiments was evaluated. In parallel a *de novo* ensemble hybrid model was developed with 1, 2, or 3 VH33 experiments, as a control simulation set. Selection of the VH33 test experiment was randomized and average performance was assessed (Figure 7).

Both the prediction error (Figure 7A,C) and the ensemble's prediction uncertainty (Figure 7B,D) were evaluated against the number of “new” VH33 experiments used for fine‐tuning. Therefore, the potential efficiency gains afforded by transfer learning approaches could be evaluated. Out of the two methods considered herein, retraining the entire ensemble at a lower learning rate (Figure 7C) outperformed retraining only the outer layer (Figure 7A) in terms of predictive capability in unseen experiments. It is highly probable that the increased degrees of freedom combined with the ability to retrain the first layer which is linked to the One Hot Encoded categorical variables enable more efficient adaptation of the ensemble to new strain‐dependent effects. However, these observations do not support a universally applicable methodological conclusion and further context‐specific research into transfer learning approaches is necessary. Moreover, in the context of the datasets considered in the present work, the benefits of transfer learning approaches compared to the development of a bespoke *de novo* hybrid model substantially diminished once all three available experiments were added to the (re‐)training set. However, it is worth noting that while prediction error from the *de novo* model was comparable from as little as two training datasets, prediction uncertainty remained higher compared to the transfer learning approach until all three datasets were included for model training (Figure 7D). This is particularly interesting, considering the fact that the transfer learning approach involves an ensemble of 28 models while the *de novo* trained ensemble comprised just three models. These results indicate that the model pretrained on DH5α data had internalized fundamental, strain‐transcendent bioprocess dynamics. Consequently, this pre‐acquired knowledge was efficiently transferred and specialized for the novel VH33 strain, validating the premise that meaningful biological principles were learned and could be generalized to new fermentation scenarios.

### 3.3 Transfer Learning: Predicting Fed‐Batch Process Performance From Batch Data

**Figure 8:**
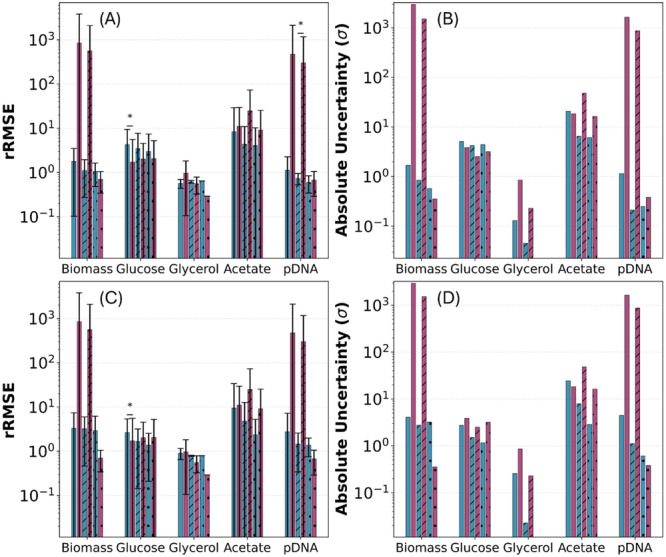
Prediction quality for unseen fed‐batch cultures. (A and C) Mean relative RMSE (rRMSE) and (B and D) absolute uncertainty of prediction using a: (i) transfer learning approach with an ensemble hybrid model pretrained on batch culture data (blue bars) and (ii) a *de novo* ensemble hybrid model trained only on fed‐batch data (purple bars). Bar patterns represent the number of experiments used for model (re‐) training which can be one (clear bars), two (dashed bars), or three (dotted bars) experimental datasets. Statistical comparisons conducted via Mann–Whitney U tests with asterisks denoting significant differences (*p < 0.05 and **p < 0.01). Transfer learning applied via (A and B) retraining the last layer in the ANN and (C and D) retraining all layers of the ANN at a reduced learning rate.

Another common process‐related change is the transition from a batch to a fed‐batch process and/or intensification of an existing fed‐batch process. This commonly occurs during the transition from early to late process development primarily due to the fact that high‐throughput experimental systems are preferentially operated in batch mode, to reduce resource demands and operational complexity (Łącki 2014). An ensemble hybrid model was developed using exclusively experiments in conducted in batch mode (Exp. 1–14; Table 1). Subsequently, the model's ability to predict an unseen fed‐batch experiment (Exp. 15–18; Table 1) when (i) the outer‐layer of the ANN was fine tuned (Rogers et al. 2022) (Figure 8A,B) or (ii) the entire ensemble was finetuned at a lower learning rate (Figure 8C,D) with 1, 2, or 3 additional fed‐batch experiments was evaluated. In parallel a *de novo* ensemble hybrid model was developed with 1, 2, or 3 fed‐batch experiments, as a control simulation set. Selection of the fed‐batch test experiment was randomized and average performance was assessed (Figure 8).

The benefits of using a transfer learning approach were more pronounced when considering the transition from batch to fed‐batch processes. Both transfer learning approaches substantially outperformed the *de novo* hybrid model until all three experimental datasets were included for (re‐) training. Retraining of the outer layer only was marginally superior to retraining the full model at a reduced learning rate (Figure 8A,C). In contrast to the analysis in Section 3.2, no new categorical variables were introduced which in turn diminished the benefits of a full network retraining to the extent where both transfer learning approaches had virtually identical performance. Absolute prediction uncertainty for the *de novo* models (three model ensemble) remained high even when all three experiments were included while the transfer learning approach (132 model ensemble) was consistently lower irrespective of the number of experiments used for model retraining. This demonstrates that the transfer learning approach not only improves prediction accuracy but also greatly enhances the model's predictive capabilities when extrapolating to fed‐batch operation, effectively mitigating the high variance typically associated with data‐limited training scenarios. The model pretrained on batch data was able to provide good quality predictions with as little as one additional experimental dataset. Therefore, transfer learning approaches in this context can significantly reduce the amount of costly and time‐consuming fed‐batch experiments required to develop an accurate model.

## 4 Conclusions

The production of pDNA is a critical yet complex bioprocess, characterized by highly nonlinear dynamics that are significantly influenced by host strain selection, the properties of the propagated plasmid, media composition, environmental process parameters (such as pH and aeriation), and fermentation mode. Herein, the development and evaluation of a robust ensemble hybrid modeling framework that integrates deep learning kinetic rate predictors with mechanistic dynamic mass balance equations was presented. The presented hybrid modeling approach leverages the structural guarantees of “first principles” equations while harnessing the flexibility of data‐driven approaches to learn from highly diverse experimental data. The model's ability to yield reliable predictions across a wide range of unseen batch fermentation conditions was demonstrated. In addition, a progressive transfer learning strategy to substantially accelerate model adaptation to new process conditions was presented and evaluated. The benefits of utilizing historic data to gain process knowledge, at a fraction of the computational, and crucially experimental, cost were demonstrated on two industrially relevant case studies: (a) migration to a new strain or cell line and (b) transition from batch to fed‐batch fermentation. In both cases, a transfer learning based approach led to significantly improved or equivalent predictive capabilities at a fraction of the experimental data, although more research on the selection of transfer learning methodology is still necessary.

The present study underscores that hybrid modeling approaches, augmented with transfer learning, are powerful tools for expediting bioprocess development. They not only enhance predictive capability across diverse conditions but also directly address the industry's need to reduce the time and cost associated with extensive experimental campaigns. Future work will focus on expanding the model's applicability to a broader range of bioprocesses and its ability to robustly operate as part of model‐predictive control strategies.

## Author Contributions

**Nikolaos Stratis:** writing – review and editing, writing – original draft, software, methodology, investigation, formal analysis, data curation, conceptualization. **Massimo Morbidelli:** writing – review and editing, methodology, investigation, conceptualization, funding acquisition, supervision, project administration. **Alexandros Kiparissides:** writing – review and editing, methodology, investigation, validation, supervision, project administration, formal analysis, conceptualization.

## Conflicts of Interest

The authors declare no conflicts of interest.

## Supporting information

Supporting File

Supporting File

## Data Availability

The dataset used in this study is available in Supporting File S5. A demonstration of the implemnetation in Python can be accessed at https://github.com/alex-kip/pDNAhybridmodels.
