# Supplementary material for: On the Prediction of pDNA Productivity Across Diverse Bioprocesses Using Ensemble Hybrid Models
Source: Biotechnol Bioeng. 2026 May 13;123(7):1771–82. doi: 10.1002/bit.70207 (PMC13309247; doi:10.1002/bit.70207)
Supplement: Supplementary file 1 — Supporting File [file BIT-123-1771-s002.docx]

**Supplementary Material**

**On the prediction of pDNA productivity across diverse bioprocesses using ensemble hybrid models**

Nikolaos Stratis^1^, Massimo Morbidelli^1^, Alexandros Kiparissides^1*^

^1^Department of Chemical Engineering

Aristotle University of Thessaloniki

54124, Thessaloniki, Greece

**S1. Inlet flowrates for fed-batch cultures**

All fed-batch experiments considered herein (Exp. 15-18; Table 1) employ an exponential feeding strategy. Hence, the volumetric flowrate of the inlet stream (*F_in_*) was calculated by [1], [2]:

$F_{in}(t)=\frac{\mu_{setpoint}\cdot V_{0}\cdot X_{0}}{S_{in}\cdot Y_{x/s}} \cdot e^{\mu\cdot t} (\frac{L}{h})$ (Eq. S1)

where $\mu_{setpoint}$ is the target growth rate ($h^{-1}$), $V_{0} \left( L \right)$ and $X_{0} (g_{CDW})$ are the initial volume and biomass at the start of the feed, respectively, and $S_{in} (\frac{g}{L})$ is the substrate concentration in the feeding solution. $Y_{x/s} (\frac{g_{CDW}}{g})$ is the yield of biomass per glucose in grams.

**S2. ANN architecture and tuning**

The ANN initially employs a normalized linear layer ($f_{NLL}$) to map inputs ($\hat{{\hat{\boldsymbol{\theta}}}_{\boldsymbol{t}}}\boldsymbol{=}\left( \vec{x}_{t},\vec{dx}_{H},\vec{v}_{OHE} \right)$) to the hidden dimension. The hidden dimension comprises rectified linear unit (ReLU) activation functions ($f_{RLU}$) embedded within linear layers, forming nested linear-ReLU blocks. Finally, the outer layer uses a softplus function ($f_{s}=ln\left( 1+e^{x} \right)$) embedded within a linear layer to ensure strictly positive predictions and smooth behavior for small input values, promoting stable learning. *The final ANN architecture, obtained through Bayesian hyperparameter optimization, consists of two hidden layers with 22 neurons each and a history length of H = 6 past time steps used in the input representation. This configuration is comprised of a total of 1123 parameters for each model.*

$\hat{r}_{t}= f_{s}\left( \boldsymbol{w}_{\boldsymbol{3}}\cdot f_{RLU}\left( \boldsymbol{w}_{\boldsymbol{2}}\cdot f_{RLU}\left( f_{NLL}\left( \boldsymbol{w}_{\boldsymbol{1}}\cdot{\hat{\boldsymbol{\theta}}}_{\boldsymbol{t}}+\boldsymbol{b}_{\boldsymbol{1}} \right) \right)+\boldsymbol{b}_{\boldsymbol{2}} \right)+\boldsymbol{b}_{\boldsymbol{3}} \right)$ (Eq. S2)

where $w_{n}$ and $b_{n}$ are learnable weights and biases respectively.

The adaptive optimization algorithm ‘adam’ [3] was employed for model development and tuning. Initially an aggressive learning rate (1e^-2^) is employed, while a learning rate scheduler (‘ReduceLROnPlateau’) is activated close to convergence to avoid overshooting. The initial learning rate of the optimizer was set 1e^-2^ allowing for an aggressive kickstart. The learning rate scheduler monitors validation loss and automatically reduces learning rate when stagnation is detected, facilitating finer learning dynamics as training progresses toward a minimum.​​ The ANN-ODE hybrid model is integrated using the Euler method. Each experiment sequence is processed to predict dynamic profiles over time, with backpropagation performed via autograd utility for all network parameters. At each optimization step, gradient clipping is applied to the calculated gradients. This involves enforcing a pre-defined maximum L2 norm of 1.0 to ensure numerical stability and prevent gradients from exploding. *The ANN input initialization procedure uses a zero padding strategy for the first H historical derivative values. Additionally, the predicted states are recursively fed back as ANN inputs for rate estimation at each subsequent time step.*

To avoid overfitting and promote generalization, validation loss is tracked alongside training loss, and model selection is carried out based on minimal combination of training and validation loss. After training, model pruning is applied with a ratio set to 20%, reducing model ensemble complexity while maintaining predictive performance. This step discards the least performant 20% of models (by validation loss), preserving a more robust and efficient representation for downstream use.​

The transfer learning approach was performed using two distinct methodologies. In the first, the parameters of the last layer in the ANN of the pretrained model ($\boldsymbol{w}_{\boldsymbol{3}}\boldsymbol{,}\boldsymbol{b}_{\boldsymbol{3}}$) were re-estimated by adding one, two or three additional experiments at a time. In the second, the parameters of all layers ${{\boldsymbol{(}\boldsymbol{w}_{\boldsymbol{1}}\boldsymbol{,}\boldsymbol{b}_{\boldsymbol{1}}\boldsymbol{,w}}_{\boldsymbol{2}}\boldsymbol{,}\boldsymbol{b}_{\boldsymbol{2}}\boldsymbol{,w}}_{\boldsymbol{3}}\boldsymbol{,}\boldsymbol{b}_{\boldsymbol{3}}\boldsymbol{)}$ were re-estimated, again by adding one, two or three additional experiments at a time. In both methodologies the learning rate was initiated at 1e-3, which was an order of magnitude lower than the initial learning rate of the pretrained model. Lastly, to produce the transfer learning models, pretrained models were fine tuned for one-twentieth of the epochs, with scheduling managed from ReduceLROnPlateau as before.

***S3. Hybrid Model performance (dynamic profiles)***


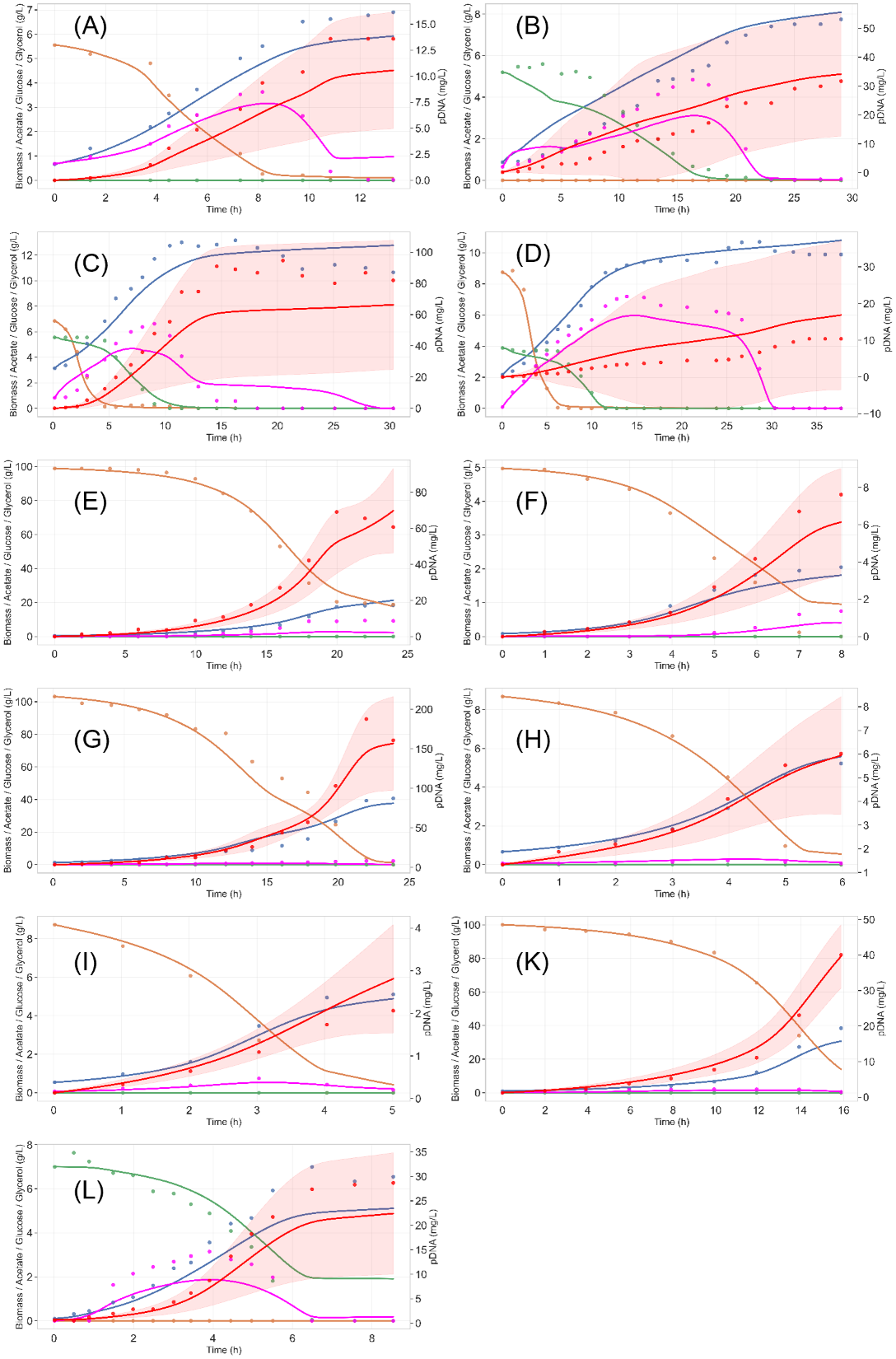


***Figure S1:*** *Simulated (–) and experimental (•) values for all state variables across all unseen datasets: (A) Exp. 1, (B) Exp. 2, (C) Exp. 3, (D) Exp. 5, (E) Exp. 6, (F) Exp. 7, (G) Exp. 8, (H) Exp. 10, (I) Exp. 11, (K) Exp. 13, (L) Exp. 14. Data for Biomass concertation (blue), Glucose concentration (light orange), Glycerol concentration (green), Acetate concentration (magenta) and pDNA concentration (red, secondary Y axis). The shaded region represents ±1 standard deviation from the ensemble mean. For visual clarity, prediction uncertainty is shown only for this variable.*

***S4. Coefficients of determination for train and test sets***

***
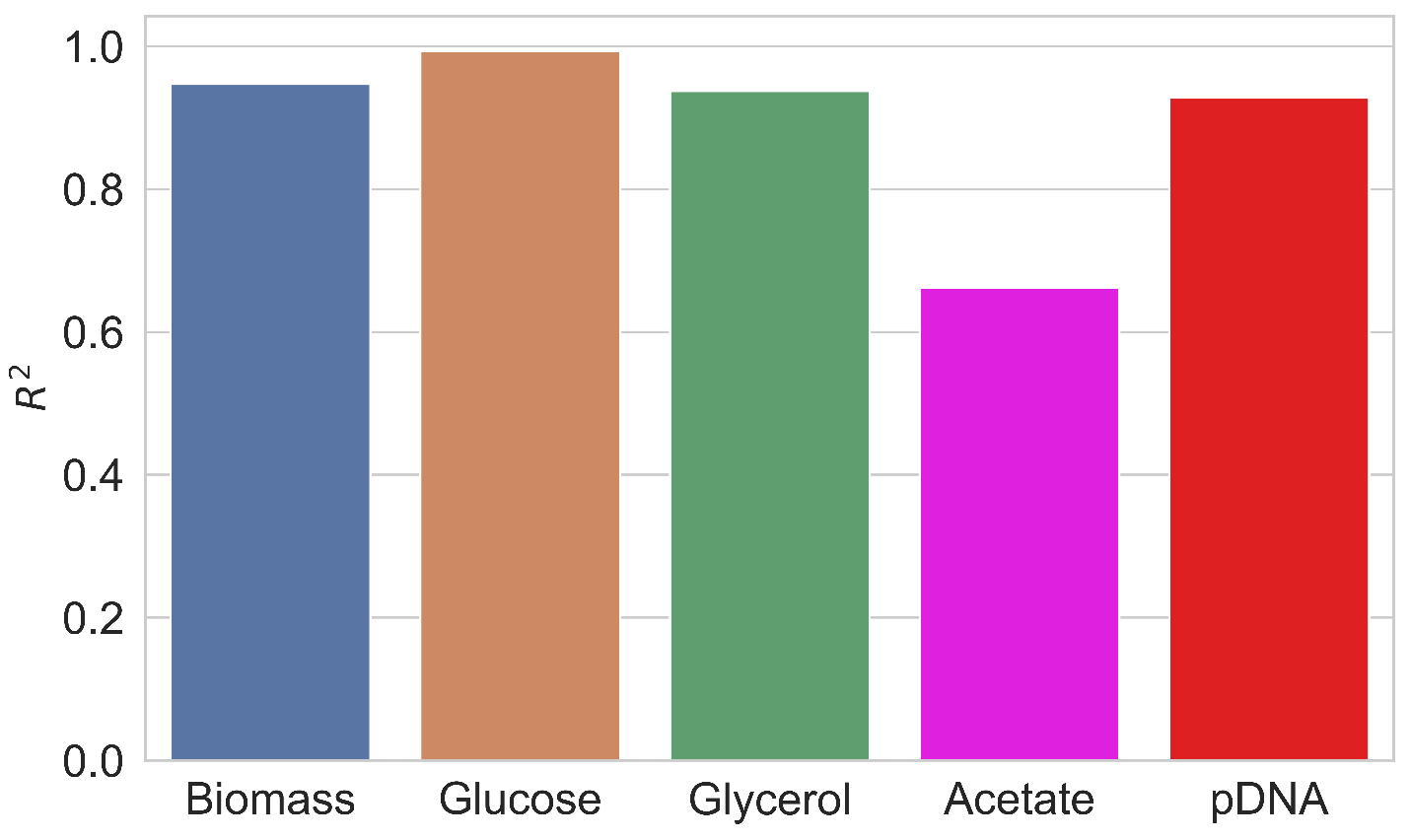
***

***Figure S2:*** *Overall coefficient of determination (R^2^) for the training subset (Exp. 1,2,3,5,6,7,8,10,11,13,14).*

***
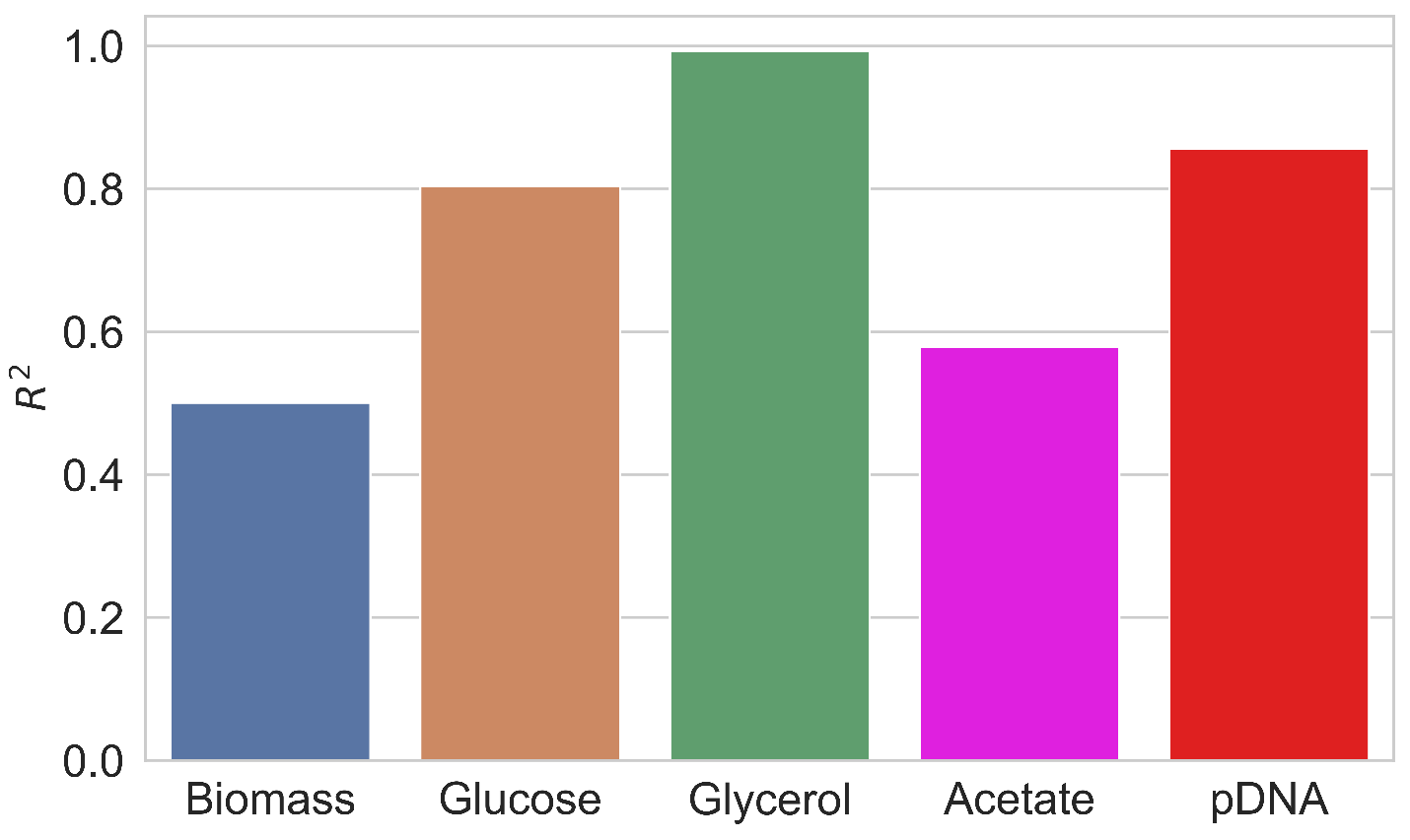
***

***Figure S3:*** *Overall coefficient of determination (R^2^) for the test subset (Exp. 4,9,12).*

**References**

[1] R. Soto, L. Caspeta, B. Barrón, G. Gosset, O. T. Ramírez, and A. R. Lara, “High cell-density cultivation in batch mode for plasmid DNA production by a metabolically engineered E. coli strain with minimized overflow metabolism,” *Biochem Eng J*, vol. 56, no. 3, pp. 165–171, Oct. 2011, doi: 10.1016/j.bej.2011.06.003.

[2] D. S. Ow, M. G. Yap, and S. K. Oh, “Enhancement of plasmid DNA yields during fed‐batch culture of a fruR‐knockout *Escherichia coli* strain,” *Biotechnol Appl Biochem*, vol. 52, no. 1, pp. 53–59, Jan. 2009, doi: 10.1042/BA20070260.

[3] D. P. Kingma and J. Ba, “Adam: A Method for Stochastic Optimization,” Jan. 2017, [Online]. Available: http://arxiv.org/abs/1412.6980
